# Direct Valorization of Biogas Residue: A Comparative Study on Facile Chemical Modifications for Superior Adsorption of Anionic Dyes

**DOI:** 10.3390/toxics14010064

**Published:** 2026-01-09

**Authors:** Xin Luo, Wenxia Zhao, Lin Fu, Yun Deng, Weijie Xue, Changbo Zhang, Ian Beadham, Zhongyan Lu, Yuyao Liu, Fanshu Bi, Qingshuai Wang

**Affiliations:** 1 Key Laboratory of Original Agro-Environmental Pollution Prevention and Control, Tianjin Key Laboratory of Agro-Environment and Agro-Product Safety, Agro-Environmental Protection Institute, Ministry of Agriculture and Rural Affairs (MARA), Tianjin 300191, China; l_xin01@163.com (X.L.); fl1004197187@163.com (L.F.); tzfelicity@163.com (W.X.); 15590811177@163.com (Y.L.); bfs1650930622@outlook.com (F.B.); wangqs512@163.com (Q.W.); 2 School of Environment and Ecology, Jiangnan University, Wuxi 214122, China; cicizhao@sinbon.com; 3 Hubei Key Laboratory of Biomass Resource Chemistry and Environmental Biotechnology, School of Resource and Environmental Sciences, Wuhan University, Wuhan 430079, China; 4 School of Pharmacy and Chemistry, Kingston University, Kingston Upon Thames KT1 2EE, UK; i.beadham@kingston.ac.uk; 5 Guangxi Tongyuan Information Technology Co., Ltd., Nanning 530200, China; luzhongyan666@sina.com

**Keywords:** biogas residue, congo red, adsorption, adsorption kinetics, adsorption isotherm

## Abstract

This study aims to develop a cost-effective and scalable modification strategy for valorizing lignin-rich biogas residue (BR) into high-performance adsorbents for anionic dye removal. To screen the optimal modification pathway, three distinct reagents, L-cysteine-based amino acid ionic liquids (AAILs, as green alternatives), conventional hydrochloric acid (HCl) and sodium hydroxide (NaOH, as traditional modification reagents), were compared in modifying non-carbonized BR for Congo Red (CR) adsorption. Comprehensive characterizations and adsorption tests revealed that each modifier exerted unique effects: NaOH only caused mild surface etching with limited performance improvement; AAILs achieved moderate adsorption capacity via a green, mild route; while HCl modification (BR-HCl) stood out with the most superior performance through a “selective dissolution-pore reconstruction” mechanism. Notably, despite a modest specific surface area increase to 12.05 m^2^/g, BR-HCl’s high CR adsorption capacity (120.21 mg/g at 45 °C) originated from the synergy of chemical bonding and enhanced electrostatic attraction—its isoelectric point (pH_PZC_ ≈ 9.02) was significantly higher than that of AAIL- and NaOH-modified samples, enabling strong affinity for anionic CR across a wide pH range. BR-HCl attained over 99% CR removal at a dosage of 0.4 g/L, fitted well with Langmuir isotherm and pseudo-second-order kinetic models (confirming monolayer chemisorption), and retained 82% of its initial capacity after five regeneration cycles. These results demonstrate that while AAILs show promise as green modifiers and NaOH serves as a baseline, the facile, low-cost HCl modification offers the most pragmatic pathway to unlock BR’s potential for sustainable wastewater treatment.

## 1. Introduction

The escalating crisis of global water scarcity, exacerbated by rapid industrialization, poses a severe threat to ecological security and socio-economic development [1]. Among various industrial effluents, textile wastewater containing refractory organic dyes represents a critical environmental hazard [2]. Congo Red (CR), a typical benzidine-based anionic diazo dye, is widely used but difficult to degrade naturally. Its discharge into water bodies not only obstructs light penetration and disturbs aquatic ecosystems but also poses significant health risks to humans, including mutagenicity and carcinogenicity, due to the formation of aromatic amines [3,4]. Therefore, the remediation of CR-contaminated water is an urgent priority.

Currently, various technologies such as chemical oxidation [5], membrane filtration [6], and photocatalytic degradation [7] have been employed for dye removal. However, these methods often suffer from critical limitations, including high operational costs, generation of toxic secondary sludge, and substantial energy consumption [8,9,10]. In contrast, adsorption is recognized as a promising water treatment technology due to its high efficiency and operational simplicity [11]. However, its industrial application is constrained by high adsorbent costs and regeneration difficulties [12,13]. Thus, developing low-cost, high-performance adsorbents from abundant, accessible raw materials with simple preparation processes is critical for promoting adsorption technology industrialization [14].

The rapid expansion of the bioenergy industry has led to a substantial increase in the production of biogas residue (digestate), primarily derived from lignocellulosic agricultural wastes such as corn, wheat, and rice straw [15]. Although biogas residue has inherent advantages for adsorbent development—including a porous structure and active functional groups—it is still largely disposed of via landfilling or used as low-value fertilizer. This not only constitutes a severe waste of resources but also poses risks of environmental pollution [16].

To date, most research on biogas residue (BR) for adsorption applications has focused on its conversion into biochar through carbonization [17,18]. However, the high energy input required for high-temperature pyrolysis inevitably compromises the economic competitiveness of the resulting adsorbents [19]. In contrast, studies on the direct modification of non-carbonized BR remain scarce, yet demonstrate promising potential for sustainable valorization. For example, Pan et al. [20,21] developed carboxylated and amine-functionalized non-carbonized BR that achieved maximum adsorption capacities of 76.92 mg/g for Cu^2+^, 64.12 mg/g for nitrate and 34.40 mg P/g for phosphate. Despite these advances, a systematic evaluation of non-carbonized modification strategies, particularly for the removal of anionic organic contaminants, is notably absent.

To achieve the goal of low-cost modification, the impregnation method, given its operational simplicity, low cost, and ease of scaling up, should be considered primarily. From the perspective of Green Chemistry, the selection of modification reagents is crucial. Strong acids or alkalis (e.g., hydrochloric acid (HCl), sodium hydroxide (NaOH)) are commonly employed to dissolve impurities and expose active sites in lignocellulose [22], representing the classic and cost-effective benchmarks for biomass activation, yet they often entail equipment corrosion and harsh operating conditions [23]. As a sustainable alternative, amino acid-based ionic liquids (AAILs) have emerged as “fully green” reagents due to their excellent biocompatibility, low volatility, and high atom economy [24]. Unlike mineral acids, AAILs offer a mild and non-corrosive processing environment, while theoretically retaining the capability to solubilize lignin and introduce amino functional groups [25].

Notably, a comparison of these three distinct reagents, HCl and NaOH versus an AAIL glycine hydrochloride ([Gly][Cl]), for modifying non-carbonized BR via impregnation is still lacking, particularly regarding their efficacy in adsorbing anionic azo dyes. Therefore, this study conducts a comparative investigation to evaluate the feasibility of these modification strategies for the removal of Congo Red (CR) [26]. Batch adsorption experiments are carried out to characterize CR adsorption behaviors, and adsorption kinetic models are applied to elucidate the underlying adsorption mechanisms. By employing the simple and cost-effective impregnation method, this work aims to identify the most practical and economically viable pathway for enhancing the performance of lignin-based adsorbents, thereby facilitating the high-value utilization of biogas residue.

## 2. Materials and Methods

### 2.1. Materials and Preparation of Adsorbents

Biogas residue (BR) was collected from straw anaerobic digestion in Wuxi, China. Chemicals including Congo Red (CR), NaOH, Glycine, and HCl were of analytical grade (Sinopharm Chemical Reagent Co., Ltd., Shanghai, China). ([Gly][Cl]) was synthesized in the laboratory following the protocol described by Tao [27]. Briefly, glycine and hydrochloric acid were mixed at a molar ratio of 1:1 in deionized water, and the solution was agitated for 8 h at 60 °C to obtain an aqueous ionic liquid solution with a mass fraction of 75%.

For modification, 10 g of dried BR was immersed in 200 mL of either 75% [Gly][Cl], 1.0 M HCl, or 1.0 M NaOH. The impregnation process was conducted at 25 °C under continuous stirring at 150 rpm for 12 h to ensure thorough penetration and thermodynamic equilibrium [28,29]. Finally, the treated solids were washed with deionized water until the pH reached neutral, dried at 60 °C, and labeled as BR-IL, BR-HCl, and BR-NaOH, respectively. Unmodified BR was labeled as BR0.

### 2.2. Characterization Method

The surface morphology was imaged by SEM (SU8010, Hitachi, Tokyo, Japan). The specific surface area and pore structure were determined by N_2_ adsorption–desorption at 77 K (TriStar II, Micromeritics, Norcross, GA, USA). The average pore diameter was calculated based on the desorption segment data of the N_2_ adsorption–desorption isotherm, using the BET model (Brunauer–Emmett–Teller model) as the core mathematical model. Additionally, the BJH model (Barrett-Joyner-Halenda model) was employed to analyze the hysteresis loop data of the N_2_ desorption isotherm for calculating the average pore diameter and pore size distribution. Functional groups were analyzed via FTIR (Nicolet iS50, Thermo Scientific, Waltham, MA, USA), and elemental composition (C, H, N, O) was determined using an elemental analyzer (Unicube, Elementar, Langenselbold, Germany).

### 2.3. Batch Adsorption Experiments

Adsorption experiments were conducted by adding adsorbents to 100 mL of CR solution in sealed vials, agitated at 100 rpm. The effects of dosage (0.1–1.6 g/L), pH (4–10), and co-existing ions (Cl^−^, NO_3_^−^, SO_4_^2−^, CO_3_^2−^) were investigated at 25 °C with an initial CR concentration of 200 mg/L, the pH of the solutions was adjusted to the desired values using 0.1 M HCl or 0.1 M NaOH solutions prior to the addition of adsorbents. The Zeta potential of the adsorbents was measured using a Zeta potential analyzer (Zetasizer Nano ZSP, Malvern Instruments, Malvern, UK) to determine the isoelectric point. Briefly, the adsorbent samples were dispersed in deionized water and ultrasonicated to form a uniform suspension. The pH of the suspension was adjusted from 2 to 10 using 0.1 M HCl or 0.1 M NaOH. The Zeta potential was recorded at each pH value, and the pH at which the Zeta potential was zero was identified as the isoelectric point. For kinetics, samples were withdrawn at predetermined intervals (5–600 min). For isotherms, initial concentrations ranged from 50 to 400 mg/L. This concentration range was selected to encompass typical dye levels found in industrial textile effluents (usually 10–200 mg/L) [30] while also extending to higher concentrations to evaluate the maximum adsorption capacity (*q_max_*) under shock loading conditions [31]. After filtration (0.45 μm), the residual CR concentration was quantified by UV-Vis spectrophotometry at 498 nm. The adsorption capacity (*q_e_*, mg/g) and removal efficiency (*R*, %) were calculated using standard mass balance equations. To ensure data reproducibility, all experimental runs were performed in triplicate. The results are presented as the mean values ± standard deviation (SD), and error bars have been added to all relevant figures to reflect statistical variability.

### 2.4. Isotherm Model and Kinetics

The adsorption kinetics of Congo Red onto the modified biogas residues were analyzed to determine the rate-controlling mechanism. The data were fitted to the Pseudo-First-Order [32] model, which assumes the rate is proportional to the number of free active sites, and the Pseudo-Second-Order [33] model, which assumes chemisorption is the rate-limiting step. Both models are defined by the following equations, respectively:

(1) $$q_{t}=q_{e}(1-e^{-k_{1}t})$$

(2) $$q_{t}=\frac{k_{2}q_{e}^{2}t}{1+k_{2}q_{e}t}$$

Additionally, the Intraparticle Diffusion model was applied to identify diffusion mechanisms:

(3) $$q_{t}=k_{di}t^{1/2}+c$$

where *q_t_* (mg/g) is the adsorption capacity at time *t* (min), *q_e_* (mg/g) is the equilibrium adsorption capacity, *k*_1_ (min^−1^) is the pseudo-first-order rate constant, *k*_2_ (g/mg·min) is the pseudo-second-order rate constant, *k_di_* (mg·g^−1/2^·min^−1/2^) is the intraparticle diffusion rate constant, and C (mg/g) is a constant related to the thickness of the boundary layer.

The adsorption isotherm data were analyzed to describe the distribution of dye molecules between phases. Three classical adsorption isotherm models were employed, including the Langmuir model (Equation (4), assuming monolayer adsorption on homogeneous sites) [34], the Freundlich model (Equation (5), assuming multilayer adsorption on heterogeneous surfaces) [35], and the Temkin model (Equation (6), suitable for describing adsorption behavior under medium coverage). Their mathematical expressions are as follows:

(4) $$q_{e}=\frac{K_{L}q_{max}C_{e}}{1+k_{L}C_{e}}$$

(5) $$q_{e}=K_{F}C_{e^{1/n}}$$

(6) $$q_{e}=\frac{RT}{b_{T}}\ln\left(K_{T}C_{e}\right)$$

where *q_e_* (mg/g) is the equilibrium adsorption capacity, *C_e_* (mg/L) is the equilibrium concentration, *q_max_* (mg/g) is the maximum adsorption capacity, *K_L_* (L/mg) is the Langmuir adsorption equilibrium constant, *K_F_* (mg/g·(L/mg)^1/n^) is a constant related to adsorption capacity, and *n* is a constant related to adsorption intensity, *R* (8.314 J/mol·K) is the universal gas constant, T (K) is the absolute temperature, *b_T_* (J/mol) is the Temkin constant related to the heat of sorption, and *K_T_* (L/mg) is the Temkin equilibrium binding constant.

Non-linear regression analysis was employed in this study using OriginPro 2024 software (OriginLab Corp., Northampton, MA, USA) to prevent the distortion of error structures inherent in linearization methods.

### 2.5. Regeneration Studies

Regeneration studies were performed by desorbing the saturated adsorbent with 0.1 M NaOH for 12 h, followed by washing and freeze-drying for subsequent cycles. The removal efficiency (*R*) was calculated based on the concentration difference before and after adsorption.

## 3. Results and Discussion

### 3.1. Physicochemical Properties of Modified Biogas Residues

#### 3.1.1. Physical Morphology

**Figure 1:**
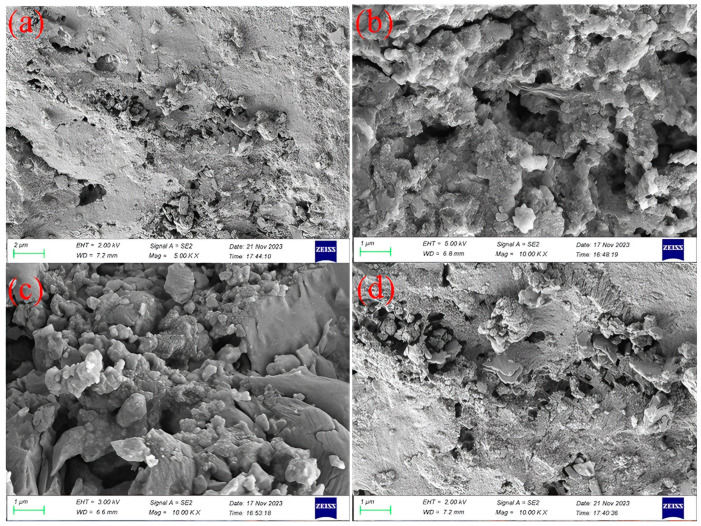
SEM image of modified biogas residue: (**a**) BR0, (**b**) BR-HCl, (**c**) BR-IL, (**d**) BR-NaOH.

To investigate the effects of different chemical modification agents on the physicochemical properties of biogas residue (BR), scanning electron microscopy (SEM) was employed to characterize the surface morphology of BR-based adsorbents before and after modification. As shown in Figure 1a, the surface of unmodified BR (BR0) showing a rough surface and irregular morphology. After modification with two acidic solutions (hydrochloric acid and ionic liquid), the surface roughness of BR-HCl (Figure 1b) and BR-IL (Figure 1c) is significantly enhanced, the structure becomes looser, and the surface exhibits more unevenness and abundant pores. This phenomenon may be attributed to the dissolution of certain components in BR by HCl and ionic liquid, which creates a rich pore structure [36]. Compared with BR0, the surface roughness and porosity of NaOH-modified BR (BR-NaOH, Figure 1d) are slightly improved, but the changes are less pronounced than those of BR-IL and BR-HCl.

The specific surface area (SSA) and pore structure parameters of different BR samples are summarized in Table 1. The SSA of unmodified BR0 is 4.14 m^2^/g. After modification, the SSAs of BR-HCl, BR-IL, and BR-NaOH increase to 12.05 m^2^/g, 11.15 m^2^/g, and 6.01 m^2^/g, respectively, with BR-NaOH exhibiting the smallest variation in pore structure parameters. The pore volumes of BR-HCl and BR-IL increase from 0.011 cm^3^/g (BR0) to 0.027 cm^3^/g and 0.018 cm^3^/g, respectively, while their average pore diameters decrease from 6.86 nm (BR0) to 6.58 nm and 6.61 nm, respectively. In contrast, the pore volume (0.010 cm^3^/g) and average pore diameter (6.63 nm) of BR-NaOH show no obvious fluctuations compared with BR0.

These differences originate from the distinct interaction mechanisms between various modifiers and BR components. BR is mainly composed of lignocellulose (cellulose, hemicellulose, lignin) and ash. During NaOH modification, NaOH only selectively acts on the amorphous regions of cellulose and the glycosidic bonds of hemicellulose, with weak degradative capacity for lignin and inability to dissolve ash (e.g., silicates, carbonates). Its effect is limited to slight surface swelling and peeling, without destroying the original pore skeleton or generating a large number of new pores. Thus, the pore structure parameters of BR-NaOH remain close to those of BR0, showing the smallest variation. In contrast, HCl and ionic liquids (AAILs) as acidic modifiers exert dual effects: on one hand, they can dissolve impurities such as small-molecule organics and calcium-magnesium salts filling the original pores of BR, exposing the previously blocked micropores; meanwhile, partial components (e.g., hemicellulose, ash) on the walls of macropores/mesopores are dissolved, leading to slight collapse and shrinkage of these pores [37]. On the other hand, under acidic conditions, lignin and hemicellulose in BR undergo partial hydrolysis and degradation, generating a large number of uniformly sized micropores (<2 nm) and small mesopores (2–5 nm) [38]. Additionally, ionic liquids can bind to BR components through intermolecular forces, inducing the rearrangement of lignin molecules to form a more regular pore structure with a concentrated pore size distribution [39]. The combined effect of the increased proportion of newly formed small pores and the shrinkage of original macropores results in a slight decrease in the average pore diameters of BR-HCl and BR-IL compared with BR0, and the pore size distribution becomes more favorable for adsorbate diffusion [40].

These results are consistent with the SEM observations, confirming that HCl and ionic liquid modification are significantly more effective in optimizing the pore structure of BR through the “selective dissolution-pore reconstruction” pathway, compared with NaOH modification which only achieves slight surface etching. Although the specific surface area (SSA) of BR-HCl increased from 4.14 m^2^/g to 12.05 m^2^/g after HCl modification, it remains relatively low compared to commercial porous carbons. However, it is worth noting that SSA is not the sole determinant of adsorption performance. This relatively low SSA implies that the efficient removal of Congo Red by BR-HCl may not primarily rely on physical pore filling, but rather on the alteration of surface chemical properties and the exposure of active sites. This hypothesis will be further corroborated in the subsequent analysis of the adsorption mechanism.

#### 3.1.2. Chemical Compositions

**Figure 2:**
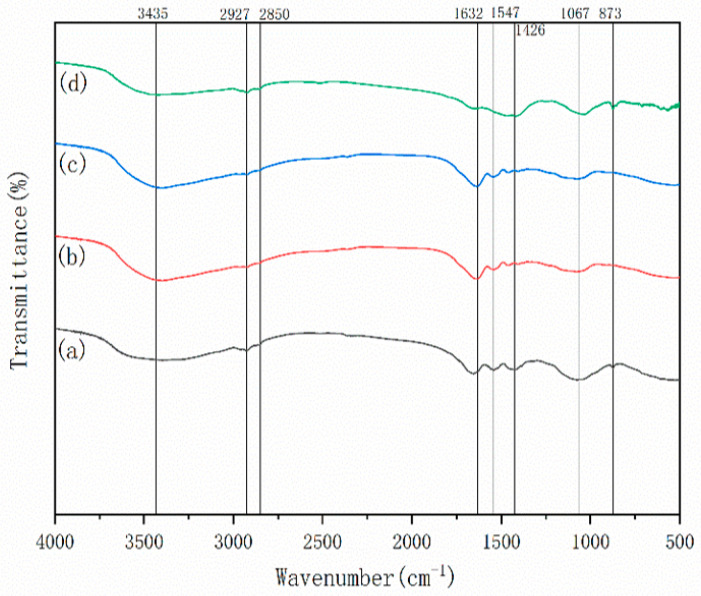
FTIR spectra of biogas residue (a) BR0, (b) BR-HCl, (c) BR-IL, (d) BR-NaOH.

The FTIR spectra of biogas residue (BR) samples are presented in Figure 2. A broad absorption band centered at 3435 cm^−1^ is assigned to the stretching vibration of -OH groups, which originates from the phenolic hydroxyl groups in lignin and alcoholic hydroxyl groups in cellulose within BR [41]. The absorption peaks at 2927 cm^−1^ and 2850 cm^−1^ correspond to the C-H stretching vibrations of -CH_3_ and -CH_2_ groups in aliphatic moieties [42]. These two functional groups (-OH and aliphatic C-H) show no obvious changes in peak position or intensity before and after modification, suggesting that the fundamental hydroxyl and aliphatic structures of BR are not destroyed by the applied modification methods.

The absorption peak at 1632 cm^−1^ is attributed to the stretching vibration of C=O groups (e.g., carbonyl, ester, or ketone groups). Notably, the peak areas of BR-HCl and BR-IL are significantly increased compared with BR0, indicating an increase in C=O content after acid and ionic liquid (AAILs) modification. In contrast, the peak area of BR-NaOH is reduced, implying that NaOH modification leads to a decrease in C=O-containing functional groups. The peak at 1547 cm^−1^ corresponds to the C-H bending vibration of methyl (-CH_3_) and methylene (-CH_2_) groups; the reduced peak area of BR-NaOH suggests partial cleavage of these aliphatic chains, while BR-HCl and BR-IL show no significant variations in this peak.

The absorption peak at 1067 cm^−1^ is associated with the C-O-C stretching vibration of the pyranose ring skeleton (typical of cellulose and hemicellulose) [43]. The peak at 1426 cm^−1^ is assigned to the aromatic ring skeleton vibration of lignin, accompanied by the in-plane C-H bending vibration and asymmetric C-H bending vibration of -CH_3_/-CH_2_ groups. For BR-HCl and BR-IL, the peak intensities at 1426 cm and 1067 cm^−1^ are obviously weakened, indicating that acidic and AAILs modification causes partial degradation or cleavage of the aromatic ring skeleton (lignin) and pyranose ring structure (cellulose/hemicellulose) in BR. In contrast, NaOH modification only results in a slight reduction in methyl/methylene-related peaks (1547 cm^−1^) without significant changes in aromatic or pyranose ring peaks, suggesting its limited effect on the main structural components of BR.

These FTIR results are consistent with the previously observed pore structure and surface morphology changes, further confirming that HCl and AAILs modification exert a more intensive effect on BR structural rearrangement and chemical composition adjustment than NaOH modification.

The discrepancy between the moderate surface area increase (12.05 m^2^/g) and the substantial structural changes observed in FTIR implies a quality-over-quantity mechanism. While AAILs theoretically introduce amino groups, the viscous nature of ionic liquids might partially block the newly formed micropores, hindering the accessibility of internal sites. In contrast, the small-molecule HCl can deeply penetrate the lignocellulosic matrix. It not only clears the pore channels via ‘selective dissolution’ but also protonates the surface functional groups more effectively [44]. This suggests that the superior performance of BR-HCl is driven by the optimization of surface chemical topology rather than the mere expansion of physical surface area, highlighting the efficiency of proton-mediated activation.

The elemental analysis results of three types of biogas residue are presented in Table 2. After modification, the carbon content of the biogas residue decreases, the oxygen content increases and the nitrogen and hydrogen contents change slightly. This further demonstrates that the modification damages the high carbon functional groups in the biogas residue and increases the oxygen containing functional groups.

### 3.2. Influence of Adsorption Process Parameters on the Removal Rate

**Figure 3:**
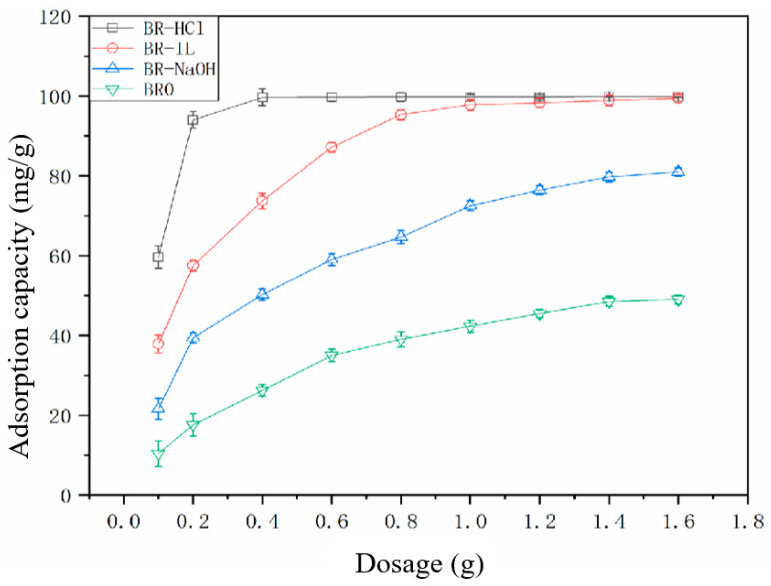
Removal of Congo Red at different dosages.

Using a Congo Red concentration of 200 mg/L and a solution volume of 100 mL, the removal rates of Congo Red by four biogas residue samples at different dosages were determined. The results are shown in Figure 3. As the dose increases from 0.1 g to 1.6 g, the removal rate of Congo Red by BR0 increases from 10.37% to 49.03%. The removal of Congo Red by BR-HCl, BR-IL and BR-NaOH increases from 59.67%, 37.85% and 21.61% to 99.81%, 99.45% and 80.99%, respectively. At the same dosage, the removal rates of Congo Red by the samples are, in descending order, BR-HCl > BR-IL > BR-NaOH > BR0. As the dosage increases from 0.1 to 0.2 g the removal rate of Congo red by BR-HCl rapidly increases from 59.6% to 94%. As the dose continues to increase, the rate of increase in removal slows. When the dosage of BR-HCl reaches 0.4 g, the removal rate is already higher than 99% and remains essentially unchanged. Meanwhile, the removal rates of Congo Red by the other three types of biogas residue continue to increase with increasing dosage.

**Figure 4:**
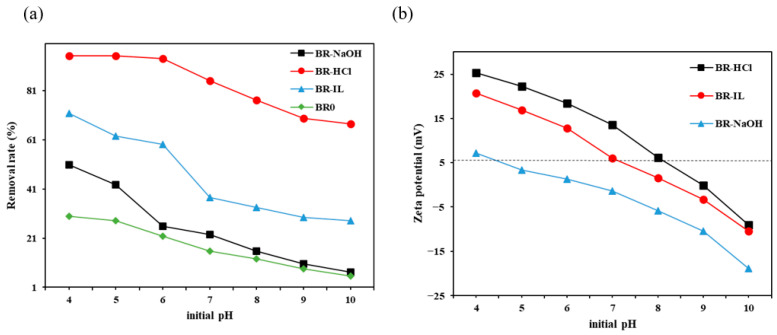
(**a**) The effect of initial pH on CR removal rate. (**b**) Zeta potential of different biogas residue. (Note: The dotted line represents the zero potential point (0 mV).).

To investigate the influence of pH on the removal rate, the removal rates of Congo Red by modified biogas residues were studied at different pH values under the conditions of a dosage of 0.4 g/100 mL and a Congo Red concentration of 200 mg/L. As can be seen from Figure 4a, the removal rates of Congo Red by the samples are, in descending order, BR-HCl > BR-IL > BR-NaOH > BR0. As the pH increases from 4 to 10, the adsorption capacities of the four types of biogas residue for Congo Red all decrease. At pH 4, the removal of Congo Red by sample BR-HCl is highest at 95.07%.

The decrease in the adsorption capacity of different biogas residues for Congo Red with increasing pH is mainly attributed to the fact that under different pH conditions, the surface properties of the adsorbent and the chemical form of the adsorbate change to different extents [45]. The isoelectric points of BR-HCl, BR-IL and BR-NaOH are 9.02, 8.37 and 6.48, respectively. (Figure 4b). The zeta potential of BR-HCl is higher than that of other adsorbents. When the pH is less than 9.02, the surface of BR-HCl is positively charged; when the pH is greater than 9.02, the surface of BR-HCl is negatively charged. The molecular structure of Congo Red contains sulphonic acid groups. Under acidic conditions, these groups are easily ionized, making the molecular structure unstable and forming soluble anions. In a lower pH environment, more H^+^ accumulates on the surface of the biogas residue, allowing the negatively charged Congo Red to combine with BR-HCl by electrostatic attraction [46]. As the pH decreases, the stronger the positive charge of BR-HCl, the stronger the electrostatic attraction between it and Congo Red. Thus, the removal rate of Congo Red by BR-HCl is significantly increased under acidic conditions. At pH greater than 5, Congo Red is present in the form of negatively charged anions. As the pH increases, the positive charge carried by BR-HCl gradually weakens and even becomes negative at pH above 9.02, resulting in electrostatic repulsion between BR-HCl and Congo Red. At the same time, in an alkaline environment, excess OH- in the solution will compete with the active sites of Congo Red, making it more difficult for Congo Red molecules to adsorb onto BR-HCl [47].

**Figure 5:**
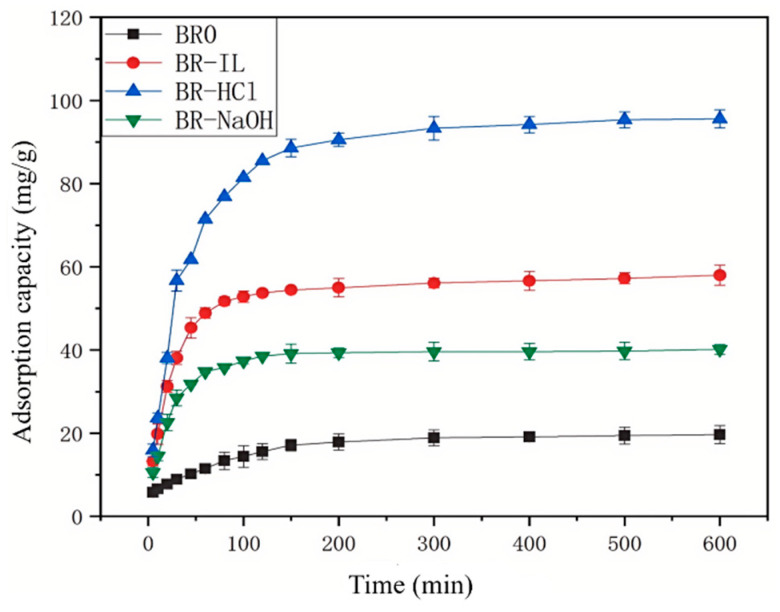
Effect of adsorption time on the adsorption effect of Congo Red.

The adsorption capacity of Congo red by biogas residues at different adsorption times is shown in Figure 5. In the initial stage of adsorption, the adsorption rates of different biogas residues are all relatively fast. Within 2 h of adsorption time, the removal efficiency can exceed 95% of the maximum removal rate. This phenomenon occurs because in the early stages of the reaction, many active sites on the surface of the adsorbent are easily and rapidly occupied by Congo red molecules. As the adsorption process time increases, the active sites on the surface of the adsorbent are gradually reduced, resulting in a continuous slowing of the adsorption rate. However, different modified biogas residues reach equilibrium at different times. The adsorption of BR0 reaches equilibrium at 500 min. The adsorption of BR-IL and BR-NaOH reaches equilibrium at 300 min. The adsorption of BR-HCl reaches equilibrium at about 200 min. The equilibrium adsorption amounts of BR0, BR-NaOH, BR-IL and BR-HCl are 19.51 mg/g, 39.59 mg/g, 56.08 mg/g and 93.31 mg/g, respectively.

To evaluate the practical applicability of the developed adsorbents in complex wastewater matrices, the effects of different coexisting anion concentrations on the adsorption of Congo Red were investigated using the three modified biogas residues. It is worth noting that the unmodified BR0 was excluded from this specific assessment due to its limited initial adsorption capacity (~19 mg/g), which renders the evaluation of ionic interference less significant for practical implementation compared to the high-performance modified samples.

**Figure 6:**
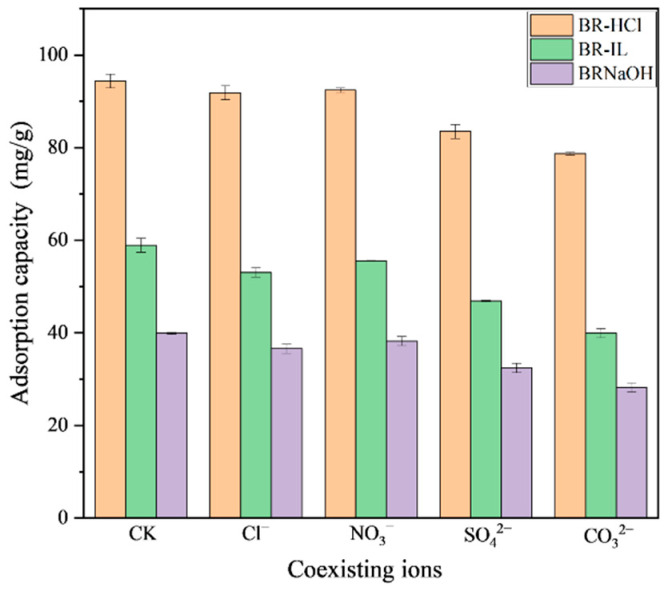
The influence of coexisting ions on the adsorption efficiency of Congo Red. (Note: “CK” stands for “Control”, representing the blank control group using deionized water without adding any competing anions.).

The influence of different concentrations of coexisting anions on the adsorption of Congo Red by the three types of adsorbents is shown in (Figure 6). In the presence of 0.1 mol/L Cl^−^ and NO_3_^−^, respectively, the adsorption capacity of Congo red by BR-HCl decreases from 94.09 mg/g to 91.54 mg/g and 92.15 mg/g. The adsorption capacity of Congo red by BR-IL decreases from 58.75 mg/g to 52.92 mg/g and 55.34 mg/g, respectively. The adsorption capacity of Congo red by BR-NaOH decreases from 39.66 mg/g to 36.20 mg/g and 38.14 mg/g, respectively. This indicates that the presence of Cl^−^ and NO_3_^−^ has minimal influence on the adsorption of Congo red by different biogas residues. The addition of SO_4_^2−^ can significantly reduce the adsorption capacity of biogas residue on Congo red. The adsorption capacity of Congo red by BR-HCl decreases from 94.09 mg per gram to 83.39 mg/g. The adsorption capacity of Congo red by BR-IL decreases from 58.75 mg/g to 46.86 mg/g. The adsorption capacity of Congo red by BR-NaOH decreases from 39.66 mg/g to 32.19 mg/g. CO_3_^2−^ has the most significant effect on the adsorption capacity. The adsorption capacity of Congo red by BR-HCl decreases from 94.09 mg/g to 78.51 mg/g. The adsorption capacity of Congo red by BR-IL decreases from 58.75 mg/g to 39.71 mg/g. The adsorption capacity of Congo red by BR-NaOH decreases from 39.66 mg/g to 28.14 mg/g.

The distinct inhibitory patterns of co-existing anions provide further insight into the adsorption mechanism. The negligible effect of monovalent ions (Cl^−^ and NO_3_^−^) confirms that ionic strength alone does not significantly suppress CR uptake [48]. However, the pronounced inhibition by multivalent anions (SO_4_^2−^ and especially CO_3_^2−^) suggests a competitive adsorption mechanism governed by charge density [49]. Since the adsorption of anionic CR relies heavily on electrostatic attraction to the positive sites on BR-HCl, anions with higher valence can compete more aggressively for these binding sites. Additionally, the hydrolysis of CO_3_^2−^ creates an alkaline micro-environment, which may locally deprotonate the surface of BR-HCl, thereby weakening the electrostatic attraction force.

### 3.3. Adsorption Kinetics

**Figure 7:**
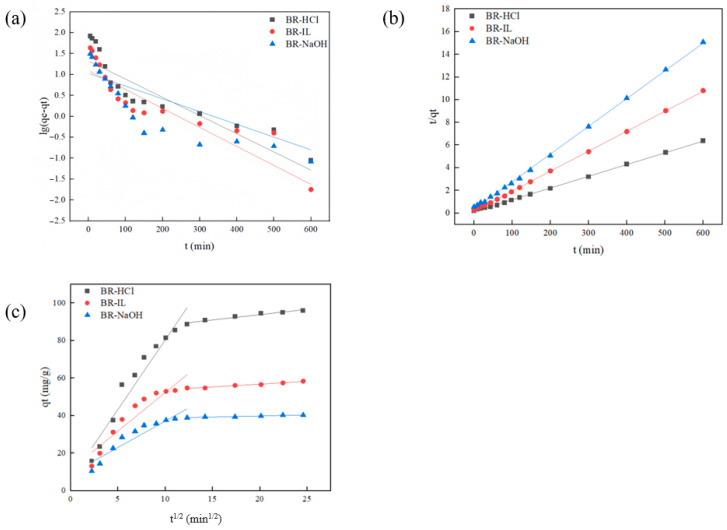
Adsorption kinetic modelling of different digestates: (**a**) Pseudo-first-order kinetics model, (**b**) Pseudo-second-order kinetics model, (**c**) intra particle diffusion model. (Note: solid lines represent the non-linear fits of the corresponding kinetic models.).

Adsorption kinetics is the key to studying the adsorption process. The removal rate and adsorption capacity of pollutants during the adsorption process can be manifested in adsorption kinetics. In addition, adsorption kinetics can provide a reliable experimental basis for an in-depth understanding of the adsorption mechanism. Through the analysis of the adsorption kinetic model, the adsorption characteristics of Congo red on biogas residues can be better understood. Figure 7a–c show the pseudo-first-order kinetic fitting curve, the pseudo-second-order kinetic fitting curve and the intraparticle diffusion model fitting curve of Congo Red dye on chemically modified biogas residue, respectively. The pseudo-first and pseudo-second-order kinetic models are used to investigate the adsorption kinetic behavior of Congo Red on chemically modified biogas residues. Figure 7a,b and Table 3 show the fitting results for the pseudo-first-order and pseudo-second-order models. The experimental equilibrium adsorption capacity (*q_e,exp_*) was significantly higher than the calculated value (*q_e,cal_*) derived from the pseudo-first-order model, and the correlation coefficient (*R*^2^) was relatively low. This indicates that the adsorption behavior of Congo Red on the modified biogas residues does not follow the pseudo-first-order model. In contrast, the experimental data exhibited a much better fit with the pseudo-second-order model, where the *q_e,cal_* values were in excellent agreement with the theoretical values, and the *R*^2^ values were close to unity (>0.99). Although kinetic models are empirical in nature, this high compliance with the pseudo-second-order model suggests that the adsorption rate is likely governed by chemisorption mechanisms involving electron sharing or exchange between the active groups on the adsorbent surface and the dye molecules, rather than being solely controlled by physical interactions.

To further elucidate the diffusion mechanisms and rate-limiting steps, the intraparticle diffusion model was applied to fit the experimental data [50]. As illustrated in Figure 7c, the plots of *q_t_* versus t^1/2^ exhibit multilinearity, indicating that the adsorption process involves distinct kinetic stages. The process can be divided into two linear regions. The first stage (0–80 min) corresponds to external surface adsorption (or instantaneous adsorption), characterized by a higher rate constant (*k_d_*_1_) and a lower intercept (*C*_1_). This rapid uptake is driven by the high initial concentration of Congo Red and the abundance of accessible active sites. As the external sites became saturated and the concentration gradient decreased, the process entered the second stage, corresponding to intraparticle diffusion. In this phase, dye molecules are diffused into the internal pores of the modified residues. Consequently, the adsorption rate decreased (*k_d_*_2_ < *k_d_*_1_), and the effect of the boundary layer became more pronounced, as reflected by a larger intercept (*C*_2_) [51]. Finally, the plots reached a horizontal plateau. This final stage represents the adsorption equilibrium phase where the rates of adsorption and desorption are balanced, and thus, kinetic dynamics cease. Consequently, the curve does not pass through the origin, suggesting that intraparticle diffusion is not the sole rate-limiting step, and the overall kinetics are controlled by a combination of surface adsorption and intraparticle diffusion mechanisms [52].

The kinetic data also reflects the structural advantages of BR-HCl. The rapid equilibrium time (~200 min) compared to BR0 (~500 min) indicates that HCl modification effectively facilitated the mass transfer process. Although the ash content was not quantitatively monitored in this study, the significant increase in BET specific surface area (from 4.14 to 12.05 m^2^/g) and pore volume (from 0.011 to 0.027 cm^3^/g) for BR-HCl strongly suggests the effective removal of pore-blocking impurities. According to previous studies on biomass modification, acid treatment is known to dissolve inorganic minerals (ash) and hydrolyze amorphous components, thereby unclogging the pores and exposing more active sites [53]. This removal minimizes steric hindrance, allowing the bulky Congo Red molecules to access the internal active sites more freely. Furthermore, the compliance with the pseudo-second-order model suggests that the adsorption rate is likely controlled by the availability of active sites and electron-exchange interactions (chemisorption mechanism), although further thermodynamic studies would be required to definitively quantify the bond energy.

### 3.4. Adsorption Isotherm

The Congo Red adsorption isothermal characteristics of BR-HCl were analyzed using three typical isothermal models, namely the Langmuir model, the Freundlich model and the Temkin model. The Langmuir model assumes monolayer adsorption on a homogeneous surface with no interaction between the adsorbate molecules. The Freundlich model is suitable for describing the non-ideal state of multi-molecular layer adsorption on a heterogeneous surface. The Temkin model considers the interactions between the adsorbent and the adsorbate, assuming that the heat of adsorption of all molecules in the layer decreases linearly with coverage.

**Figure 8:**
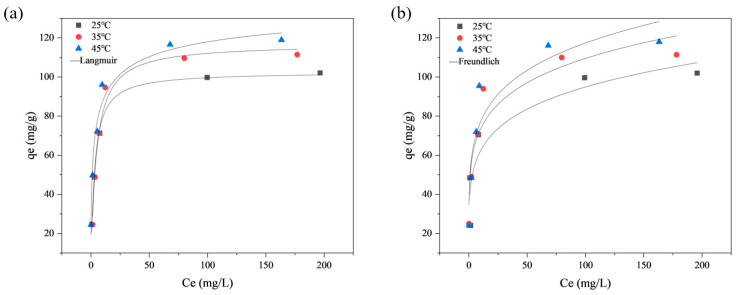
Adsorption isotherm modelling of BR-HCl: (**a**) Langmuir, (**b**) Freundlich.

The results are shown in Figure 8, and the parameters are listed in Table 4. Under the adsorption temperatures of 25 °C, 35 °C and 45 °C, the correlation coefficients of the Langmuir isotherm model fitting are 0.9694, 0.9829 and 0.9773, respectively, which are consistently higher than the correlation coefficients of the Freundlich isotherm model fitting (0.7461, 0.7742, 0.7963). This implies that the adsorption process of BR-HCl on Congo red is more accurately fitted by the Langmuir isotherm model. This conformity suggests that the adsorption occurs as a uniform monolayer coverage on specific active sites. Furthermore, as suggested by the strict monolayer assumption, this behavior is often indicative of strong specific interactions (chemisorption) rather than non-specific physical multilayer accumulation. The maximum adsorption capacities obtained by fitting the Langmuir isotherm model are 105.82, 114.12 and 120.21 mg/g. Furthermore, the equilibrium data were analyzed using the Temkin isotherm model to gain insights into the energy distribution. The Temkin model exhibited high correlation coefficients (*R*^2^) ranging from 0.9253 to 0.9634 across all temperatures. This good fit suggests that the adsorption heat of Congo Red molecules decreases linearly with coverage due to adsorbent-adsorbate interactions. The variation in the Temkin constant *b_T_* (137.15–210.43 J/mol) indicates a non-uniform distribution of binding energies on the BR-HCl surface, which is consistent with the rough pore structure observed in SEM images.

A notable phenomenon observed in this study is the apparent mismatch between the relatively low specific surface area of BR-HCl (12.05 m^2^/g) and its remarkably high adsorption capacity (120.21 mg/g). Generally, physical adsorption relies heavily on high porosity and large surface area. The contradiction here provides compelling evidence that the adsorption of Congo Red onto BR-HCl is governed by chemisorption and specific interactions rather than non-specific physical pore filling.

First, electrostatic attraction plays a pivotal role. As indicated by the Zeta potential analysis, BR-HCl possesses a high isoelectric point (pH_PZC_ ≈ 9.02). Under the experimental conditions (pH < 9), the highly positively charged surface of BR-HCl exerts a strong electrostatic attraction towards the anionic sulfonate groups of Congo Red molecules. This allows for substantial dye uptake even without a highly developed pore structure.

Second, the kinetic and isotherm modeling results support the dominance of chemical adsorption. The experimental data fitted perfectly with the pseudo-second-order kinetic model (*R*^2^ = 0.9995) and the Langmuir isotherm model. The adherence to the Langmuir model specifically implies that the dye molecules occupy specific fixed sites on the adsorbent surface through monolayer adsorption, a characteristic typical of chemisorption processes involving strong intermolecular forces (e.g., electron sharing or exchange) [54]. Moreover, the high Temkin constant *b_T_* and binding constant K_T_ further corroborate the existence of strong binding forces between the adsorbent and pollutant. Furthermore, the FTIR analysis revealed an increase in oxygen-containing functional groups (e.g., C=O), which likely serve as binding sites for the dye molecules through hydrogen bonding or coordination, compensating for the limitation in surface area.

### 3.5. Comparison with Other Adsorbents

To evaluate the potential application of BR-HCl in practical wastewater treatment, its maximum adsorption capacity (*q_max_*) for Congo Red was compared with other adsorbents reported in the literature (Table 5). As shown in the table, BR-HCl exhibits a significantly higher adsorption capacity (120.21 mg/g) compared to many unmodified agricultural wastes and is competitive with some synthetic composites. This suggests that BR-HCl is a promising and cost-effective candidate for the removal of anionic dyes.

### 3.6. Reusability Studies

**Figure 9:**
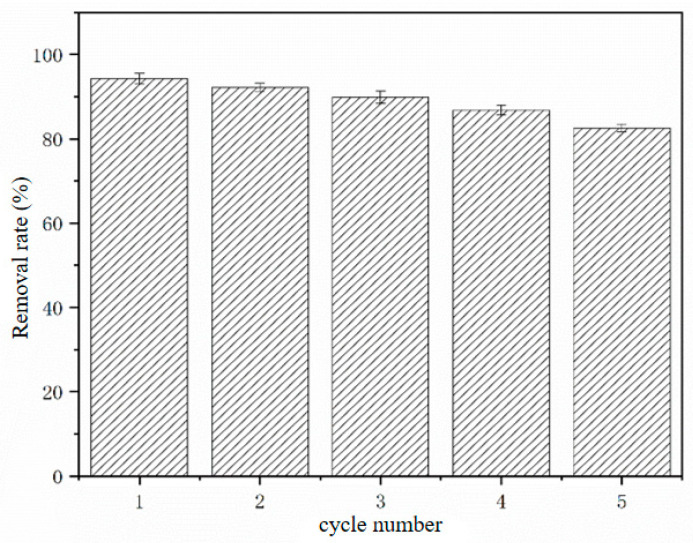
Cyclic regeneration adsorption performance.

In current production applications, the recycling time of adsorbents can have a significant impact on adsorption efficiency and the cost of adsorbent preparation. Among the prepared materials, BR-HCl has the best adsorption performance for Congo Red dye. Therefore, BR-HCl is selected in this study to investigate the influence of cycle number on the adsorption capacity. The results show that the adsorption efficiency of BR-HCl decreases as the number of cycles increases (Figure 9). Possible reasons for the analysis are as follows: during each adsorption–desorption process of the BR-HCl absorbent, the loss of surface functional groups leads to a decrease in the number of functional groups on the surface of the BR-HCl absorbent. In addition, during the multiple adsorption–desorption process, the residual Congo Red on the surface of the BR-HCl adsorbent will also cause a reduction in the number of active sites. In addition, during the number of adsorption–desorption cycles, some of the remaining Congo Red molecules on the surface of the adsorbent will occupy the active sites, resulting in a decrease in the adsorption efficiency of the adsorbent. However, after 5 cycles of regeneration, the adsorption efficiency of BR-HCl for Congo Red decreases by only 18% compared to the initial adsorption efficiency. This indicates that BR-HCl has good adsorption-regeneration properties.

Although the regeneration performance is robust, the observed ~18% decline in capacity after five cycles warrants attention. This attenuation is likely due to the irreversible chemisorption of dye molecules within the deep micropores, where strong hydrogen bonding or entrapment prevents complete desorption by NaOH. Furthermore, repeated exposure to alkaline regeneration solutions may cause partial leaching of the low-molecular-weight lignin or hemicellulose components from the biogas residue skeleton. Nevertheless, the retention of >80% capacity confirms the structural stability of BR-HCl for long-term industrial applications.

## 4. Conclusions

This comparative study successfully establishes a facile and cost-effective pathway for valorizing lignin-rich biogas residue (BR) into efficient adsorbents for anionic dye removal. The results demonstrate that a simple HCl impregnation method (BR-HCl) outperforms both conventional NaOH and emerging amino acid ionic liquid (AAIL) modifications, achieving a superior Congo Red adsorption capacity of 120.21 mg/g. The exceptional performance of BR-HCl is attributed to a unique “selective dissolution-pore reconstruction” mechanism. This process not only optimizes the pore structure but, more importantly, significantly elevates the surface positive charge (pH_PZC_ ≈ 9.02), thereby enabling strong electrostatic attraction and chemisorption towards anionic dyes. This synergistic mechanism effectively compensates for the material’s limited specific surface area (12.05 m^2^/g).

Furthermore, BR-HCl exhibits excellent practical potential, achieving over 99% dye removal at a low dosage and retaining 82% of its initial capacity after five regeneration cycles. While AAILs offer a greener and milder modification route, and NaOH serves as a baseline, this work conclusively shows that the HCl-mediated activation provides the optimal balance of performance, cost, and scalability. The findings challenge the assumption of the inherent superiority of complex green solvents and highlight a critical trade-off in sustainable technology development: pragmatic, acid-based treatments can sometimes deliver a more immediately viable and economically feasible solution for the valorization of agricultural waste in wastewater treatment.

## Figures and Tables

**Table 1:** Pore structure parameters of unmodified and modified biogas residue.

Sample	BET Specific Surface Area (m^2^·g^−1^)	Pore Volume (cm^3^·g^−1^)	Average Pore Diameter (nm)
BR0	4.14	0.011	6.86
BR-HCl	12.05	0.027	6.58
BR-IL	11.15	0.018	6.61
BR-NaOH	6.01	0.010	6.63

Note: “Average pore diameter” calculated using the BJH desorption method.

**Table 2:** The main element content of unmodified and modified biogas residue.

Material	Element Content			
	C (%)	H (%)	N (%)	O (%)
BR0	44.18	4.96	5.24	24.02
BR-HCl	40.12	4.89	5.12	28.27
BR-IL	41.45	4.92	5.19	26.84
BR-NaOH	42.76	4.81	5.15	25.68

**Table 3:** Adsorption kinetics equation parameters.

Material	Pseudo-First-Order Kinetics Model				Pseudo-Second-Order Kinetics Model			
	*q_e,exp_* (mg/g)	*q_e,cal_* (mg/g)	*k*_1_ (mg/(g·min))	*R* ^2^	*q_e,exp_* (mg/g)	*q_e,cal_* (mg/g)	*k*_2_ (mg/(g·min))	*R* ^2^
BR-HCl	94.036	5.9602	0.0052	0.9571	94.036	100.3009	0.0003	0.9995
BR-IL	57.476	3.9519	0.0041	0.8489	57.476	58.8928	0.0011	0.9997
BR-NaOH	39.436	2.8950	0.0038	0.8385	39.436	41.0172	0.0019	0.9996
Material	Intra particle diffusion model							
	*q_e,exp_* (mg/g)	*C*_1_ (mg/g)	*K_d_*_1_ (mg/(g·min^1/2^))	*R* ^2^	*q_e,exp_* (mg/g)	*C*_1_ (mg/g)	*K_d_*_1_ (mg/(g·min))	*R* ^2^
BR-HCl	94.036	2.5134	8.1885	0.9472	94.036	76.4213	0.860	0.8230
BR-IL	57.476	8.1410	4.7153	0.9015	57.476	50.1567	0.323	0.9677
BR-NaOH	39.436	6.9345	3.2157	0.9013	39.436	36.9543	0.136	0.7170

**Table 4:** Adsorption isotherm equation parameters.

	Langmuir Model				Freundlich Model		
	*R* ^2^	K_L_	Qm (mg/g)		*R* ^2^	K_F_	1/n
25 °C	0.9694	0.2992	105.8204		0.7461	44.5807	0.1727
35 °C	0.9829	0.3166	114.1245		0.7742	45.8774	0.1888
45 °C	0.9773	0.3458	120.2169		0.7943	48.0825	0.1940
	**Temkin Model**						
	*R* ^2^	K_T_	*b_T_*				
298.15 K	0.9416	9.86	168.28				
308.15 K	0.9253	4.21	137.15				
318.15 K	0.9634	89.47	210.43				

**Table 5:** Comparison of maximum adsorption capacities (*q_max_*) of Congo Red by various adsorbents.

Adsorbent	*q_max_* (mg/g)	Reference
BR-HCl (This study)	120.21	Present study
*Spathodea campanulata* biochar	59.27	[55]
Rice husk activated carbon	11.84	[55]
Neem leaf powder	41.2–28.3	[56]
cellulose	74.87	[57]
Bamboo Hydrochars	75.16	[3]
Magnetic fly ash	154.00	[58]
Nanozeolite X	110.24	[58]
Mycelial pellets	316.46	[59]
MgAl-LDH	769.23	[60]
Macauba palm cake	32.00	[61]

## Data Availability

Dataset available on request from the authors.
